# Identifying resident care areas for a quality improvement intervention in long-term care: a collaborative approach

**DOI:** 10.1186/1471-2318-12-59

**Published:** 2012-09-25

**Authors:** Lisa A Cranley, Peter G Norton, Greta G Cummings, Debbie Barnard, Neha Batra-Garga, Carole A Estabrooks

**Affiliations:** 1 Faculty of Nursing, University of Alberta, Level 3 Edmonton Clinic Health Academy, 11405 87 Avenue, Alberta, Edmonton, T6G 1C9, Canada; 2 Department of Family Medicine, University of Calgary, Calgary, Alberta, Canada, Mailing address: 56 George Street, Creemore, Ontario, L0M 1G0, Canada; 3 Quality and Patient Safety, Health Sciences North, 41 Ramsey Lake Road, Sudbury, Ontario, P3E 5J1, Canada; 4 Strategy and Knowledge, Community Treatment and Support, Addiction and Mental Health, Alberta Health Services, #210, 10909 Jasper Avenue, Associated Engineering Plaza, Edmonton, Alberta, T5J 3M9, Canada

**Keywords:** Quality improvement, Healthcare providers, Quality care, Long-term care

## Abstract

**Background:**

In Canada, healthcare aides (also referred to as nurse aides, personal support workers, nursing assistants) are unregulated personnel who provide 70-80% of direct care to residents living in nursing homes. Although they are an integral part of the care team their contributions to the resident care planning process are not always acknowledged in the organization. The purpose of the Safer Care for Older Persons [in residential] Environments (SCOPE) project was to evaluate the feasibility of engaging front line staff (primarily healthcare aides) to use quality improvement methods to integrate best practices into resident care. This paper describes the process used by teams participating in the SCOPE project to select clinical improvement areas.

**Methods:**

The study employed a collaborative approach to identify clinical areas and through consensus, teams selected one of three areas. To select the clinical areas we recruited two nursing homes not involved in the SCOPE project and sampled healthcare providers and decision-makers within them. A vote counting method was used to determine the top five ranked clinical areas for improvement.

**Results:**

Responses received from stakeholder groups included gerontology experts, decision-makers, registered nurses, managers, and healthcare aides. The top ranked areas from highest to lowest were pain/discomfort management, behaviour management, depression, skin integrity, and assistance with eating.

**Conclusions:**

Involving staff in selecting areas that they perceive as needing improvement may facilitate staff engagement in the quality improvement process.

## Background

The Translating Research in Elder Care (TREC) program is a multi-year study (2007–2012) whose goal is to improve the care in the long-term residential care environment (nursing homes) in Canada [1]. The major work in TREC is focused upon identification of modifiable aspects of organizational context associated with delivery of best care to residents. To carry this out TREC has collected survey data twice between 2008 and 2010 and also accessed the Resident Assessment Instrument – Minimum Data Set 2.0 (RAI-MDS) data from the sample nursing homes from 2007 onward. Survey data has been collected from both regulated and unregulated staff (e.g., healthcare aides, nurses) working in 36 nursing homes in the three prairie provinces (Alberta, Saskatchewan, Manitoba). Protocols for this project are published [1-3]. In Canada, healthcare aides (HCAs) (also referred to as nurse aides, personal support workers, nursing assistants) are unregulated personnel who provide 70-80% of direct bedside care to residents living in nursing homes. Although HCAs are an integral part of the care delivery team survey findings have indicated that their contributions to the resident care planning process are not always valued or acknowledged in the organization and that HCAs wanted to be asked for their opinions and to have their voices heard. Consequently as part of the research program the research team sought funding for and planned the Safer Care for Older Persons [in residential] Environments (SCOPE) project, with the goal of engaging and empowering front line staff to become involved (and take ownership) in a quality improvement process at the bedside. The result was SCOPE, a pilot project (2010–2012) that enabled HCA-led quality improvement teams to improve care for residents (Health Canada contribution agreement #6804-15-2009/9180076). SCOPE employed a collaborative approach to quality improvement based on similar models developed by the Institute for Healthcare Improvement for their Breakthrough Series [4] and by the *Safer Healthcare Now*! Canadian initiative [5]. The Breakthrough Series Collaborative is a shared learning system that brings teams of caregivers together with the support of content and quality improvement experts, to improve their work on focused care areas [4]. The goal of the *Safer Healthcare Now!* campaign is to use quality improvement methods to integrate evidence and best practices into direct patient care. Both employ, as a core component, the Model for Improvement [6] which addresses three questions:

1. What are we trying to accomplish?

2. How will we know that a change is an improvement?

3. What changes can we make that will result in improvement?

Changes are tested using the Plan-Do-Study-Act (PDSA) cycle of rapid change. The complete SCOPE protocol has been published elsewhere [7].

The research team recruited seven nursing homes in Western Canada, each of which agreed to the following: having one or more unit-based teams led by a HCA; naming a senior sponsor (e.g., Director of Care, Facility Manager) for each organization's team(s); providing release time (approximately 5 to 10% of a HCA position) for project related activities; and, providing financial support (up to $3,000) for staff member attendance at the learning sessions. The seven homes identified a total of 10 teams.

A critical next step was identification of a number of clinical areas for improvement. This paper describes the process used by teams participating in the SCOPE project to select clinical improvement areas.

## Methods

The University of Alberta Institutional Review Board and the Interior Health region of British Columbia Research Ethics Board approved the study protocol(#Pro00012517).

After the SCOPE teams and senior sponsors had been identified the next step was to identify a list of potential clinical areas. It was important that there was at least one area of interest for each of the SCOPE quality improvement teams and their sponsors. Hence the list should be fairly short. In a two stage process a preliminary list was developed meeting the following criteria:

1. The performance in the area is measurable using the Resident Assessment Instrument – Minimum Data Set 2.0 (RAI-MDS) system.

2. There is a known gap in care – i.e., performance was not maximal.

3. There is an evidence base and real life examples of opportunities to improve care.

4. Key stakeholders in the sector including HCAs believed the areas were important targets for improvement at the bedside.

After this list had been developed it was reviewed by the SCOPE teams and their senior sponsors to ensure that one or more areas were places they believed they could make a difference. Finally each SCOPE team selected select an individual area under the condition that a selected area would have at least two committed teams in order that collaboration and sharing could occur within a healthy air of competition. Informed consent was obtained from stakeholders by way of their participation in ranking the list of care domains.

The approach used was similar to a modified Delphi technique [8] where consultations are made with experts using both a survey and a physical meeting with the goal of achieving a consensus [9]. However, our approach varies from the modified Delphi technique in that face-to-face meetings were held not only with experts but with front line staff who provide care to residents, including SCOPE study participants (quality improvement teams). The collaborative approach used in this study also differs from other rating procedures such as the RAND appropriateness method, which combines expert opinion and available scientific evidence [10], and the nominal group technique [11,12]. The nominal group method involves a meeting to rate items, discussion and a re-rating of items [9].

In this study, quality improvement teams were involved in the process of selecting what they considered were areas relevant to their practice. Quality improvement teams were included early in the project as a means of increasing ‘buy in’ and ownership of the project, which are key aspects to engaging staff in a quality improvement project. The collaborative method used in this study incorporates a ‘Mode 2’ approach to knowledge production and translation, where knowledge is produced in the context of application [13-15]. Mode 2 knowledge production involves collaborative relationships with stakeholders, and is based on the needs of end-users [15].

To arrive at a preliminary list that satisfied points 1–3 expert consultation was employed. Then to accomplish point 4 feedback was sought from key stakeholders in the sector to refine the list.

The details of the expert consultation are as follows. The SCOPE team, informed by the TREC study data and literature on RAI-MDS 2.0 quality indicators [16], developed the preliminary list of possible areas: (1) pain management, (2) falls and restraints, (3) assistance with eating, (4) end of life care, (5) mobility, (6) medication safety, (7) continence, (8) behaviour, and (9) skin integrity. This was presented to two gerontology experts who worked clinically and as researchers in the sector together with criteria 1–3 during a face-to face meeting between them and the SCOPE research team. They suggested deletion of end of life care and medication safety because these were not as immediately relevant to HCAs’ core scope of practice. They also suggested adding two additional care areas: depression and urinary tract infection. Following their suggestions we generated a new list of ten areas of care with associated RAI-MDS indicators (Table 1).

**Table 1:** Original list of areas of care domains

** Plain language**	**RAI MDS 2.0 Quality Indicator**
1. Falls	Prevalence of falls
2. Restraints	Prevalence of daily physical restraints
3. Behaviour management	Prevalence of behaviour symptoms affecting others
4. Depression	Prevalence of symptoms of depression
5. Continence	Prevalence of bladder/ bowel incontinence
6. Bladder infection	Prevalence of urinary tract infection
7. Eating/feeding/nutrition	Prevalence of weight loss Prevalence of dehydration
8. Mobility	Prevalence of little or no activity ^a^
9. Skin integrity/skin care	Prevalence of pressure ulcers
10. Pain/discomfort management	Prevalence of pain (frequency and intensity)

Note. We removed falls from the list sent to healthcare aides after we learned that *Safer Healthcare Now!* was potentially launching a national initiative on falls in the long-term care sector.

^a^ There was no quality indicator for mobility at the time this list was sent to stakeholders.

The next (second) stage involved input from stakeholder groups. There were four stakeholder groups: (1) senior decision-makers; (2) registered nurses/care coordinators; (3) managers/educators; and (4) HCAs. Each was charged with prioritizing the elements in the original list considering both the relevance and importance for quality improvement from their perspective. The following techniques were used to solicit this information.

### Senior decision-makers

A member of the SCOPE research team emailed the list of quality indicators to two of the TREC program’s senior decision-makers who were not directly involved with SCOPE and asked them to rank the top five RAI-MDS 2.0 quality indicators that aligned with corporate objectives as areas for improvement.

### Registered Nurses/Care Coordinators and Managers/Educators

A SCOPE research team member was invited to attend an infection control meeting with registered nurses/care coordinators, managers and educators from the sector. During the meeting the SCOPE team member asked the attendees to identify which clinical topics from the original list were of interest to them if they were to work on an improvement project in the long-term care sector.

### Healthcare aides

SCOPE project personnel led facilitated discussions in two nursing homes (not SCOPE sites) in different provinces. These discussions began with the distribution of a paper copy of the plain language part of Table 1 to a group of HCAs. The SCOPE project team discussed the list with HCAs who were then asked to select up to five areas of care they were most interested in improving in their unit.

The final round of consultation was with the SCOPE quality improvement teams in the seven SCOPE project sites. It began with the SCOPE project manager holding a teleconference with these teams. Prior to the teleconference the teams had received the material presented in Table 1. The need to shorten the list to three was discussed. Then the teams were asked to meet privately together with their senior sponsor to select the area they planned to work on during 12 months of the SCOPE project and to communicate it back to the SCOPE team within a week.

## Results

**Figure 1:**
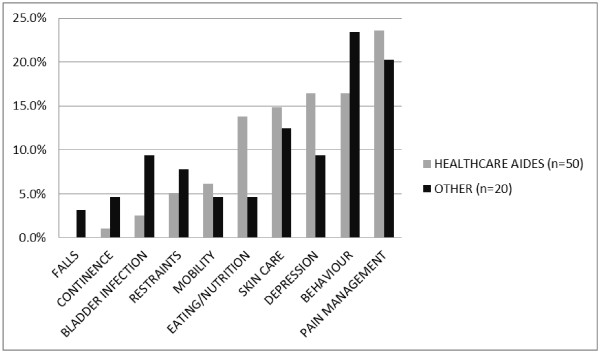
Percent of total votes for each criterion by group.

In the second round of consultation, responses were received from 50 HCAs, two senior decision-makers, four registered nurses/care coordinators, and 14 managers/educators. The HCAs cast 195 votes or a mean of 3.9 votes per HCA. The 20 remaining stakeholders are categorized into a group called Other and cast 64 votes (mean of 3.2). The percentage of total votes for each clinical area by HCAs and Other are shown in Figure 1. The top five priority areas of care for improvement in order of highest ranked were: (1) pain/discomfort management, (2) behaviour management, (3) depression, (4) skin integrity, and (5) assistance with eating.

In the final round the quality improvement teams selected the following areas of care to focus on improving: four teams selected pain management, four teams selected behaviour management, and two teams chose skin care.

## Discussion

This report describes an efficient but engaged process to select a small number of quality improvement areas for our project. The entire process took less than two weeks and accomplished the main goals that are outlined in the criteria above. In the end the quality improvement teams and their senior sponsors had no problem in selecting an area of interest to them from the ranked items.

The similarities in rankings of the HCAs and other stakeholders are of interest and in fact the top five areas were the same for both groups. Restraints and bladder infection appear to be more important to the Other group than to the HCAs while eating and nutrition and mobility were more important to HCAs than to Others. There is no data from this exercise to explain these differences but one can speculate that HCAs who assist residents with meals and their mobility have a different perspective on the problems associated with these activities than those with less resident contact during these occasions. On the other hand, in nursing homes in the project, provincial policy limits the use of restraints and this type of policy limitations may have more direct impact on the Other group than the HCAs. As well, HCAs may believe that urinary tract infections are a normal part of the aging process while Others are concerned about the subtle effect these may have on survival and costs.

The differences noted within the top five rankings between HCAs and the Other group may be associated with differences in their scope of practice. It may be that the Other group (e.g., nurses, educators) selected areas where they believe they can have a significant impact. Over the past decade there has been a growing literature on nursing-sensitive outcomes. Nursing-sensitive outcomes are described as those that are “relevant, based on nurses’ scope and domain of practice, and for which there is empirical evidence linking nursing inputs and interventions to the outcome” [17]. In the long-term care sector, there is evidence that two nursing-sensitive outcomes, functional status (independence in activities of daily living) and symptom control (pain), are correlated with several nursing interventions [18]. For instance, Doran et al. [18] found that pain frequency and severity were related to nursing interventions for analgesic administration.

In the long-term care setting, HCAs provide the majority of essential care to older persons living in nursing homes [19] and are key to the quality of care delivered in nursing homes. HCAs work under the supervision of a regulated health care provider (typically a registered nurse) and are responsible for providing assistance with personal care activities, such as mobilizing, dressing, bathing, toileting, grooming, and eating. HCAs have detailed knowledge of residents (e.g., biographical and vocational histories) [20] and their own personal experiences and capabilities which contribute to their understanding of residents’ behaviour, the individualizing of care (person-centered care) [20,21], and their use of best practices [21]. Indeed, HCAs contribute to resident outcomes within their scope of practice. For instance, Corazzini and colleagues [22,23] found that when HCAs participated in decisions about resident care, their involvement in the decision-making process was related to improved outcomes in urinary tract infections.

This project has limitations. First, it was carried out in two jurisdictions in Canada so it is not possible to correct for differences that may exist in other jurisdictions due to the models, policy decisions, staffing patterns and the like. Second, since SCOPE was designed to be a pilot project, and in fact to act as proof-of-principle, the sample of nursing homes chosen was a convenience one. Therefore further testing would be necessary if one wished to apply this method to other samples.

## Conclusions

This approach to selecting areas for quality improvement was efficient and useful. Involving staff in selecting areas of resident care they perceive as needing improvement is a first step to facilitating staff engagement and thus “buy in” in the quality improvement process. It also enables the targeting of improvement areas that are perceived to be relevant to direct care providers and the residents for whom they provide care. This last feature is important to enhancing the likelihood of adoption success [24].

## Abbreviations

TREC: Translating Research in Elder Care; HCA: Healthcare aides; SCOPE: Safer Care for Older Persons [in residential] Environments; RAI-MDS 2.0: Resident Assessment Instrument- Minimum Data Set 2.0.

## Competing interests

The authors declare that they have no competing interests.

## Authors' contributions

CAE and PGN conceived of the project and secured funding for the project and participated in the project design and coordination. LAC and NBG conducted participant recruitment, data collection and analysis. LAC drafted the manuscript. CAE, PGN, GGC, NBG and DB provided feedback on the draft manuscript. All authors read and approved the final manuscript.

## Pre-publication history

The pre-publication history for this paper can be accessed here:

http://www.biomedcentral.com/1471-2318/12/59/prepub
